# Potential of Artificial Intelligence in Evidence-Based Practice in Nursing

**DOI:** 10.1590/0034-7167.2024770501

**Published:** 2024-09-09

**Authors:** Isabelle Cristinne Pinto Costa, Alice Silva Costa, Karina Dal Sasso Mendes, Ricardo Limongi

**Affiliations:** I Universidade Federal de Alfenas. Alfenas, Minas Gerais, Brazil; II Universidade de São Paulo. Ribeirão Preto, São Paulo, Brazil; III Universidade Federal de Goiás. Goiânia, Goiás, Brazil

Evidence-based practice (EBP) has established itself as a fundamental pillar in nursing, driving effective clinical decision-making based on high-quality scientific research. The primary goal of EBP is to ensure that patients receive the most appropriate and safe care, based on the best available evidence. In this context, knowledge synthesis methods are essential tools for EBP, as they contribute to clinical decision-making based on robust reviews, resulting from the combination of several studies in the more than 28,000 scientific articles published in health annually. However, the current scientific panorama is characterized by a massive production of knowledge, making the task of synthesizing and interpreting evidence a Herculean challenge for healthcare professionals. With these challenges in mind, artificial intelligence (AI) emerges as a powerful tool, capable of revolutionizing EBP and making it more efficient and accurate, advancing the time and quality of research.

AI, with its ramifications in machine learning (ML) and natural language processing (NLP), provides techniques capable of processing and analyzing colossal volumes of data, including the vast scientific literature. Some AI tools, such as Elicit, Consensus, Litmaps, Perplexity, Semantic Scholar, ResearchRabbit, Paper Digest, Scholarcy and Open Knowledge Maps, have already mapped more than 280,000 scientific articles and, based on ML, promise to revolutionize the identification of knowledge gaps. Imagine AI combing through thousands of articles, revealing unexplored areas and outlining new frontiers for nursing research, freeing up precious time for researchers to dedicate themselves to robust investigations. With this evolution, literature mapping and relevance, such as listing articles and elaborating research problems, can be discussed in real time between researchers.

Another application is in the selection of relevant articles for research, automating the screening process and prioritizing the reading of articles with the highest probability of relevance. The ASReview platform, for instance, uses ML techniques to automate article screening, prioritizing the reading of those most likely to be relevant. This functionality speeds up the selection of studies that will make up part of the review, allowing researchers to focus on the critical analysis of the most promising studies and also highlight the most important sections of the studies in an exploratory stage.

AI also emerges as an ally in the precise delimitation of study populations, which is extremely important to guarantee the validity and applicability of research results. Tools such as Litbaskets allow analyzing the representativeness of journals in relation to a specific topic, guiding researchers in choosing relevant data sources. Furthermore, AI can help optimize research protocols, improving experimental design and minimizing time and costs. Consensus, for instance, indicates studies that confirm or refute possible practices and also categorizes articles based on the methodological approach, facilitating the systematization of knowledge through different methodological approaches.

Data collection, a traditionally laborious step in review research, also benefits from the power of AI. Tools such as SourceData (EMBO), ChatGPT, ChatPDF, EnagoRead and Gemini (Google) can automatically extract relevant information from articles, reports and images, significantly facilitating and speeding up this process.

When synthesizing and interpreting evidence, AI proves to be an indispensable tool. Document grouping techniques, such as those explored in the study^(1)^, make it possible to visualize the relationship between different articles, facilitating the identification of key themes and the construction of knowledge maps. AI can also assist in the efficient communication of research results, generating concise and informative reports. Its ability to analyze complex data, such as genetic and sensor information, opens up a range of opportunities to uncover hidden patterns and generate innovative insights, assisting in formatting the scientific article and optimizing the structure, language and style. Tools like Paperpal can be used to check the clarity and cohesion of the text as well as ensuring that it follows publishing standards.

Additionally, it is essential to remain open and flexible in the face of new technologies, especially in relation to AI. We should not restrict ourselves to a single tool. It is important to explore, test and adopt solutions that best adapt to our specific needs, ensuring a broader and more effective approach to applying EBP in nursing. However, it is important to keep in mind that AI does not replace the researcher’s judgment and expertise; its reflection must start from the following understanding: AI is a means, not an end. As powerful as it is, it needs to be used ethically and judiciously, always prioritizing research quality and patient safety. Human curation, critical validation of results and accurate interpretation of conclusions generated by AI are non-negotiable pillars to guarantee evidence reliability and applicability^(2)^.

AI ushers in a new era for EBP in nursing and healthcare, opening up a horizon of possibilities. Innovative tools, driven by it, are transforming research, accelerating the discovery of new interventions and contributing to building a healthier future for all. The scientific community needs to actively engage in the debate on AI use, exploring its benefits, challenges, establishing responsible guidelines and practices for its application.

Nursing, a leader in promoting health and well-being, has the responsibility to take advantage of this transformative potential of AI, driving a more robust, efficient and impactful EBP.
