# Supplementary material for: Potential of Artificial Intelligence in Evidence-Based Practice in Nursing
Source: Rev Bras Enferm. 2024 Sep 9;77(5):e770501. doi: 10.1590/0034-7167.2024770501 (PMC11383434; doi:10.1590/0034-7167.2024770501)
Supplement: Translation

# Translation: Potencial de la Inteligência Artificial en la Práctica de Enfermería Basada en la Evidencia

**DOI:** 10.1590/0034-7167.2024770501es

**Authors:** Isabelle Cristinne Pinto Costa, Alice Silva Costa, Karina Dal Sasso Mendes, Ricardo Limongi

**Affiliations:** I Universidade Federal de Alfenas. Alfenas, Minas Gerais, Brasil; II Universidade de São Paulo. Ribeirão Preto, São Paulo, Brasil; III Universidade Federal de Goiás. Goiânia, Goiás, Brasil

La práctica basada en la evidencia (PBE) se ha consolidado como un pilar fundamental en enfermería, impulsando una toma de decisiones clínicas eficaz basada en investigaciones científicas de alta calidad. El objetivo principal de la PBE es garantizar que los pacientes reciban la atención más adecuada y segura, basada en la mejor evidencia disponible. En este contexto, los métodos de síntesis de conocimiento son herramientas esenciales para la PBE, ya que contribuyen a la toma de decisiones clínicas basadas en revisiones robustas, resultantes de la combinación de varios estudios en los más de 28.000 artículos científicos publicados anualmente en el área de la salud. Sin embargo, el panorama científico actual se caracteriza por una producción masiva de conocimiento, lo que convierte la tarea de sintetizar e interpretar evidencia en un desafío hercúleo para los profesionales de la salud. Con estos desafíos en mente, la inteligencia artificial (IA) surge como una poderosa herramienta, capaz de revolucionar la EBP y hacerla más eficiente y precisa, adelantando el tiempo y la calidad de la investigación.

La IA, con sus ramificaciones en el *machine learning* (ML) y el *natural language processing* (NLP), proporciona técnicas capaces de procesar y analizar volúmenes colosales de datos, incluida la vasta literatura científica. Algunas herramientas de IA, como *Elicit, Consensus, Litmaps, Perplexity, Semantic Scholar, ResearchRabbit, Paper Digest, Scholarcy* y *Open Knowledge Maps,* ya han mapeado más de 280.000 artículos científicos y, basándose en ML, prometen revolucionar la identificación de lagunas de conocimiento. Imagine que la IA revisa miles de artículos, revela áreas inexploradas y delinea nuevas fronteras para la investigación en enfermería, liberando un tiempo precioso para que los investigadores se dediquen a investigaciones sólidas. Con esta evolución, el mapeo y la relevancia de la literatura, como enumerar artículos y elaborar problemas de investigación, se pueden discutir en tiempo real entre investigadores.

Otra aplicación está en la selección de artículos relevantes para la investigación, automatizando el proceso de selección y priorizando la lectura de los artículos con mayor probabilidad de relevancia. La plataforma *ASReview,* por ejemplo, utiliza técnicas de aprendizaje automático para automatizar la selección de artículos, priorizando la lectura de aquellos que tienen más probabilidades de ser relevantes. Esta funcionalidad agiliza la selección de estudios que formarán parte de la revisión, permitiendo a los investigadores centrarse en el análisis crítico de los estudios más prometedores y también resaltar las secciones más importantes de los estudios en una etapa exploratoria.

La IA también emerge como una aliada en la delimitación precisa de las poblaciones de estudio, lo cual es sumamente importante para garantizar la validez y aplicabilidad de los resultados de la investigación. Herramientas como *Litbaskets* permiten analizar la representatividad de las revistas en relación a un tema específico, orientando a los investigadores en la elección de fuentes de datos relevantes. Además, la IA puede ayudar a optimizar los protocolos de investigación, mejorando el diseño experimental y minimizando tiempo y costes. La *Consensus,* por ejemplo, señala estudios que confirman o refutan posibles prácticas y también categoriza artículos con base en el enfoque metodológico, facilitando la sistematización del conocimiento a través de diferentes enfoques metodológicos.

La recopilación de datos, un paso tradicionalmente laborioso en la investigación de revisión, también se beneficia del poder de la IA. Herramientas como *SourceData* (EMBO), *ChatGPT, ChatPDF, EnagoRead*y *Gemini* (Google) pueden extraer automáticamente información relevante de artículos, informes e imágenes, facilitando y acelerando significativamente este proceso.

A la hora de sintetizar e interpretar pruebas, la IA demuestra ser una herramienta indispensable. Técnicas de agrupación de documentos, como las exploradas en el estudio^(1)^, permiten visualizar la relación entre diferentes artículos, facilitando la identificación de temas clave y la construcción de mapas de conocimiento. La IA también puede ayudar en la comunicación eficiente de los resultados de la investigación, generando informes concisos e informativos. Su capacidad para analizar datos complejos, como información genética y de sensores, abre una variedad de oportunidades para descubrir patrones ocultos y generar conocimientos innovadores, ayudando en el formato del artículo científico y optimizando la estructura, el lenguaje y el estilo. Se pueden utilizar herramientas como *Paperpal* para comprobar la claridad y cohesión del texto, así como para garantizar que sigue los estándares de publicación.

Además, es fundamental permanecer abiertos y flexibles frente a las nuevas tecnologías, especialmente en relación con la IA. No debemos limitarnos a una sola herramienta. Es importante explorar, probar y adoptar las soluciones que mejor se adapten a nuestras necesidades específicas, asegurando un enfoque más amplio y eficaz para la aplicación de la PBE en enfermería. Sin embargo, es importante tener en cuenta que la IA no reemplaza el juicio y la experiencia del investigador; su reflexión debe partir de la siguiente comprensión: la IA es un medio, no un fin. Por muy potente que sea, debe utilizarse de forma ética y sensata, priorizando siempre la calidad de la investigación y la seguridad del paciente. La curación humana, la validación crítica de los resultados y la interpretación precisa de las conclusiones generadas por la IA son pilares innegociables para garantizar la confiabilidad y aplicabilidad de la evidencia^(2)^.

La IA marca el comienzo de una nueva era para la PBE en enfermería y atención sanitaria, abriendo un horizonte de posibilidades. Las herramientas innovadoras, impulsadas por ella, están transformando la investigación, acelerando el descubrimiento de nuevas intervenciones y contribuyendo a construir un futuro más saludable para todos. La comunidad científica necesita participar activamente en el debate sobre el uso de la IA, explorando sus beneficios, desafíos, estableciendo pautas y prácticas responsables para su aplicación.

La enfermería, protagonista en la promoción de la salud y el bienestar, tiene la responsabilidad de aprovechar este potencial transformador de la IA, impulsando una PBE más robusta,

# Translation: Potencial da inteligência Artificial na Prática Baseada em Evidências em Enfermagem

**DOI:** 10.1590/0034-7167.2024770501pt

**Authors:** Isabelle Cristinne Pinto Costa, Alice Silva Costa, Karina Dal Sasso Mendes, Ricardo Limongi

**Affiliations:** I Universidade Federal de Alfenas. Alfenas, Minas Gerais, Brasil; II Universidade de São Paulo. Ribeirão Preto, São Paulo, Brasil; III Universidade Federal de Goiás. Goiânia, Goiás, Brasil

A prática baseada em evidências (PBE) consolidou-se como pilar fundamental na enfermagem, impulsionando a tomada de decisões clínicas eficazes e embasadas em pesquisas científicas de alta qualidade. O objetivo primordial da PBE é assegurar que os pacientes recebam os cuidados mais apropriados e seguros, com base nas melhores evidências disponíveis. Neste contexto, os métodos de síntese de conhecimento são ferramentas essenciais para a PBE, pois contribuem para a tomada de decisão clínica a partir de revisões robustas, resultantes da combinação de diversos estudos nos mais de 28.000 artigos científicos publicados na área da saúde anualmente. Contudo, o panorama científico atual é caracterizado por uma produção massiva de conhecimento, tornando a tarefa de sintetizar e interpretar as evidências um desafio hercúleo para os profissionais de saúde. Com esses desafios em mente, a inteligência artificial (IA) surge como uma ferramenta poderosa, capaz de revolucionar a PBE e torná-la mais eficiente e precisa, avançando em tempo e qualidade das pesquisas.

A IA, com suas ramificações em *machine learning* (ML) e *natural language processing* (NLP), proporciona técnicas capazes de processar e analisar volumes colossais de dados, incluindo a vasta literatura científica. Algumas ferramentas de IA, como *Elicit, Consensus, Litmaps, Perplexity, Semantic Scholar, ResearchRabbit, Paper Digest, Scholarcy* e *Open Knowledge Maps,* já mapearam mais de 280.000 artigos científicos e, baseadas em ML, prometem revolucionar a identificação de lacunas de conhecimento. Imaginemos a IA vasculhando milhares de artigos, revelando áreas inexploradas e delineando novas fronteiras para a pesquisa em enfermagem, liberando tempo precioso dos pesquisadores para se dedicarem às investigações robustas. Com esta evolução, o mapeamento da literatura e a relevância, como o fichamento de artigos e elaboração de problemas de pesquisa, podem ser discutidos em tempo real entre pesquisadores.

Outra aplicação é na seleção de artigos relevantes para a pesquisa, automatizando o processo de triagem e priorizando a leitura dos artigos com maior probabilidade de relevância. A plataforma *ASReview,* por exemplo, utiliza técnicas de ML para automatizar a triagem de artigos, priorizando a leitura daqueles com maior probabilidade de relevância. Essa funcionalidade agiliza a seleção dos estudos que comporão parte da revisão, permitindo que os pesquisadores se concentrem na análise crítica dos estudos mais promissores e, ainda, evidenciem os trechos mais importantes dos estudos em uma etapa exploratória.

A IA também desponta como aliada na delimitação precisa das populações de estudo, extremamente importante para garantir a validade e a aplicabilidade dos resultados de pesquisa. Ferramentas como *Litbaskets* permitem analisar a representatividade de periódicos em relação a um tema específico, guiando os pesquisadores na escolha de fontes de dados relevantes. Além disso, a IA pode auxiliar na otimização de protocolos de pesquisa, aperfeiçoando o desenho experimental e minimizando tempo e custos. A *Consensus,* por exemplo, indica estudos que confirmam ou refutam eventuais práticas e, ainda, categoriza artigos a partir da abordagem metodológica, facilitando a sistematização do conhecimento por diferentes abordagens metodológicas.

A coleta de dados, etapa tradicionalmente trabalhosa em pesquisas de revisão, também se beneficia do poder da IA. Ferramentas como *SourceData* (EMBO), *ChatGPT, ChatPDF, EnagoRead* e *Gemini* (Google) podem automaticamente extrair informações relevantes de artigos, relatórios e imagens, facilitando e agilizando significativamente esse processo.

Na síntese e interpretação das evidências, a IA revela-se uma ferramenta indispensável. Técnicas de agrupamento de documentos, como as exploradas no estudo^(1)^, permitem visualizar a relação entre diferentes artigos, facilitando a identificação de temas-chave e a construção de mapas de conhecimento. A IA pode, ainda, auxiliar na comunicação eficiente dos resultados de pesquisa, gerando relatórios concisos e informativos. A sua capacidade de analisar dados complexos, como informações genéticas e de sensores, abre um leque de oportunidades para desvendar padrões ocultos e gerar informações inovadoras, auxiliando na formatação do artigo científico e otimizando a estrutura, a linguagem e o estilo. Ferramentas como *Paperpal* podem ser utilizadas para verificar a clareza e a coesão do texto, além de garantir que ele siga as normas de publicação.

Adicionalmente, é fundamental permanecer aberto e flexível diante das novas tecnologias, especialmente em relação à IA. Não devemos nos restringir a uma única ferramenta. É importante explorar, testar e adotar as soluções que melhor se adaptam às nossas necessidades específicas, garantindo uma abordagem mais ampla e eficaz na aplicação da PBE na enfermagem. Porém, é importante ter em mente que a IA não substitui o discernimento e a *expertise* do pesquisador; a sua reflexão deve partir da seguinte compreensão: a IA é meio, não fim. Por mais poderosa que seja, ela precisa ser utilizada de forma ética e criteriosa, sempre priorizando a qualidade da pesquisa e a segurança do paciente. A curadoria humana, a validação crítica dos resultados e a interpretação acurada das conclusões geradas pela IA são pilares inegociáveis para garantir a confiabilidade e a aplicabilidade das evidências^(2)^.

A IA inaugura uma nova era para a PBE na enfermagem e na saúde, abrindo um horizonte de possibilidades. Ferramentas inovadoras, impulsionadas por ela, estão transformando a pesquisa, acelerando a descoberta de novas intervenções e contribuindo para a construção de um futuro mais saudável para todos. A comunidade científica precisa se engajar ativamente no debate sobre a utilização da IA, explorando seus benefícios, desafios, estabelecendo diretrizes e práticas responsáveis para sua aplicação.

A enfermagem, protagonista na promoção da saúde e do bem-estar, tem a responsabilidade de apropriar-se desse potencial transformador da IA, impulsionando uma PBE mais robusta, eficiente e impactante.
